# Machine learning approaches for biomarker discovery using single-cell RNA sequencing

**DOI:** 10.3389/fbinf.2026.1767362

**Published:** 2026-04-02

**Authors:** Gabriel Dewa, C. Mee Ling Munier, Sara Ballouz, Raymond Louie

**Affiliations:** 1 School of Computer Science and Engineering UNSW, Sydney, NSW, Australia; 2 Kirby Institute UNSW, Sydney, NSW, Australia

**Keywords:** artificial intelligence, biomarker discovery, feature selection, machine learning, single-cell RNA sequencing

## Abstract

The application of single-cell RNA sequencing (scRNA-seq) for biomarker discovery promises unprecedented resolution in identifying potential biomarkers by capturing and analysing cellular heterogeneity. Traditionally, biomarker discovery efforts within single-cell transcriptomics have primarily relied on conventional statistical approaches, particularly through the application of differential gene expression analysis, to identify candidate biomarkers. However, in recent years, with the rapid advancement and growing popularity of artificial intelligence and machine learning, their application in scRNA-seq biomarker discovery has become increasingly prominent. Currently, machine learning-based approaches for scRNA-seq biomarker discovery exhibit considerable methodological diversity, which can be distinguished by factors such as the level of discovery, choice of supervised learning algorithm, feature selection methods, classification metrics, and downstream biological analyses. This review provides a comprehensive overview of the current landscape of machine learning methods for scRNA-seq biomarker discovery, offering researchers a complete and detailed understanding of the field.

## 1 Introduction

A biological marker, or biomarker, is “a defined characteristic that is measured as an indicator of normal biological processes, pathogenic processes, or biological responses to an exposure or intervention, including therapeutic interventions” (FDA-NIH Biomarker Working Group, 2016). Biomarkers encompass a range of biological molecules, such as genes, proteins, and metabolites, each serving specific roles in diagnosing diseases, monitoring progression, or predicting treatment outcomes. In the era of precision medicine, validated biomarkers play an increasingly crucial role in tailoring treatments to individual patients, facilitating more accurate diagnosis, prognosis, and personalized treatment plans (Vargas and Harris, 2016). The development and validation of biomarkers is a complex, multistep, and iterative process. The initial discovery stage typically begins with a comparison of samples from different disease or condition groups to identify distinguishing biological molecules. These initial candidates are then subjected to analytical validation using statistical tests and evaluation metrics to assess reliability, reproducibility, and specificity as potential biomarkers (Kraus, 2018).

Advancements in biomarker discovery hold great promise for improving our understanding of disease mechanisms and drug development, particularly with the use of single-cell RNA sequencing (scRNA-seq). scRNA-seq enables transcriptomic profiling at the resolution of individual cells, allowing direct measurement of gene expression heterogeneity within complex tissues. By capturing transcriptional states at the single-cell level, scRNA-seq makes it possible to identify rare cell populations, characterize regulatory programs, and infer lineage relationships (Kim et al., 2021). In contrast, bulk RNA sequencing measures averaged gene expression across heterogeneous cell populations, thereby masking cell-type–specific signals and obscuring important biological variation.

This difference in resolution has important implications for biomarker discovery. In bulk RNA-seq studies, biomarker identification is typically restricted to patient-level analyses based on averaged gene expression profiles. Although such approaches can identify genes that differ between disease groups, they cannot determine whether these signals arise from specific cell types, changes in cellular composition, or coordinated transcriptional programs within particular cellular subpopulations (Kim et al., 2021).

In contrast, scRNA-seq expands biomarker discovery along three key dimensions. First, it enables a wider range of input representations, including individual cell gene expression profiles (Krokidis et al., 2023a), cell-type–specific expression signatures (Zhou et al., 2024), aggregated pseudo-bulk profiles (Kang et al., 2022), and patient-level summaries such as cell-type proportions (Dong et al., 2024). Second, this flexibility supports distinct analytical levels of discovery, namely, cell-level and patient-level machine learning frameworks, as we will describe in this review (Section 3). Third, scRNA-seq enables a broader spectrum of biomarker outputs, including disease-associated cell types, cell-type–specific gene signatures, cellular state markers, and disease subtypes defined by transcriptional or compositional heterogeneity (Krokidis et al., 2023a; Zhou et al., 2024; Kang et al., 2022; Dong et al., 2024).

Therefore, while bulk RNA-seq primarily yields patient-level gene biomarkers derived from averaged expression, scRNA-seq provides a multi-resolution framework in which cellular heterogeneity can be explicitly modelled. This expanded representation underlies the growing integration of machine learning approaches into scRNA-seq biomarker discovery, which is comprehensively examined in this review.

However, despite the rise of more affordable and higher resolution technologies such as scRNA-seq, current biomarker development continues to be hampered by low statistical power and inconsistent reproducibility of findings (Vargas and Harris, 2016). Although several factors may contribute to these challenges, one potential key contributor that affects scRNA-seq and the general field of biomarker discovery is the inherent limitations of current traditional statistical methods (Wenric and Shemirani, 2018; Zhang et al., 2021; Ng et al., 2023).

Currently, scRNA-seq biomarker discovery remains largely reliant on traditional statistical approaches, particularly differential gene expression (DGE) analysis. Albeit an effective tool, DGE analysis possess inherent limitations that can be improved to enhance its effectiveness in biomarker identification (Zhang et al., 2021). For instance, most DGE analysis tools predominantly utilize a univariate approach that conducts a significance test independently for each gene. As each gene is treated in isolation, this approach does not take into consideration potential interactions among genes, which may result in the omission of genes that individually may not be notable, but when assessed as a collective may be significant (Wenric and Shemirani, 2018; Zhang et al., 2021). Furthermore, DGE analysis use of statistical test typically requires specific distributional assumptions which cannot always be satisfied, for example, when the number of patients or number of cells are limited (Zhang et al., 2021; Ng et al., 2023).

In response to these limitations, there is a rising interest in exploring alternative approaches for biomarker discovery, particularly through the application of machine learning algorithms. In contrast to DGE analysis, machine learning approaches for biomarker discovery apply a multivariate strategy that takes into consideration potential gene interactions to identify potential biomarkers (Wenric and Shemirani, 2018; Pudjihartono et al., 2022). Additionally, machine learning algorithms offer significant flexibility and require minimal statistical assumptions, as they do not rely on predefined hypotheses or constraints (Rajula et al., 2020). By circumventing strict reliance on statistical assumptions, machine learning can also effectively capture non-linear relationships during the identification of biomarkers (Ng et al., 2023; Sabbatini and Manganaro, 2022).

The potential of machine learning over traditional DEA tools have led to a recent rise in the use of machine learning for scRNA-seq biomarker discovery. In this paper, we review the fundamental concepts and current methodologies underlying the use of machine learning in scRNA-seq–based biomarker discovery. We outline the key components and steps of a typical workflow, examine the range of available methodological approaches, and highlight their significance. Finally, we discuss important considerations for interpreting machine learning–derived biomarkers and propose potential directions for future advancement in this field.

## 2 General workflow: scRNA-seq and machine learning for biomarker discovery

Machine learning is a branch of artificial intelligence that emphasizes the study of statistical algorithms and models capable of learning underlying patterns from data and making decisions with minimal human intervention (El Naqa and Murphy, 2015). Machine learning encompasses a broad range of techniques and algorithms which can be categorized into supervised, unsupervised, semi-supervised, and reinforcement learning (Sarker, 2021a). Each machine learning category/type is based on distinct learning principles that dictate how algorithms interpret and generalize data, with each approach tailored to address specific types of challenges and tasks. In the field of single-cell transcriptomics, the use of different types of machine learning algorithms has been widely applied to address various problems. For example, the application of both unsupervised and supervised machine learning algorithms has been extensively explored for cell-type identification (Sun et al., 2022), integration (Ryu et al., 2023) and other key scRNA-seq analysis (Brendel et al., 2022). However, for the purposes of biomarker discovery, current applications of machine learning within single-cell transcriptomics primarily revolves around the application of supervised learning approaches.

Supervised learning is a machine learning approach that trains a model to understand the relationship between input features and labelled outputs. A key objective is for the model to generalize this relationship, allowing the model to predict labels of new data based on its input features. For scRNA-seq biomarker discovery, the features are typically the genes (expression), and the labels are the disease conditions. The goal is to train a supervised learning model capable of predicting a condition or disease based on gene expression profile, which can be done either at the cell or patient level (Section 3). However, not all genes contribute equally to classification. Some genes are highly informative and improve prediction accuracy, while others may be uninformative or irrelevant, acting only as noise. To ensure the best possible classification performance, it is important to only include the most relevant genes when predicting conditions. This identification of highly predictive genes that improve classification is done through a process called feature selection (Section 4.2).

Feature selection assesses the contribution of each feature to the classification performance of a machine learning model. In the context of biomarker discovery, feature selection evaluates each gene’s ability to predict different conditions. By comparing each genes’ importance and influence, feature selection can identify genes that improve accuracy while removing genes that do not contribute or reduce performance. Since feature selection only retains genes that are highly relevant for predicting different disease conditions or outcome groups, the selected genes may serve as promising potential biomarkers (Zhang et al., 2021).

**FIGURE 1:**
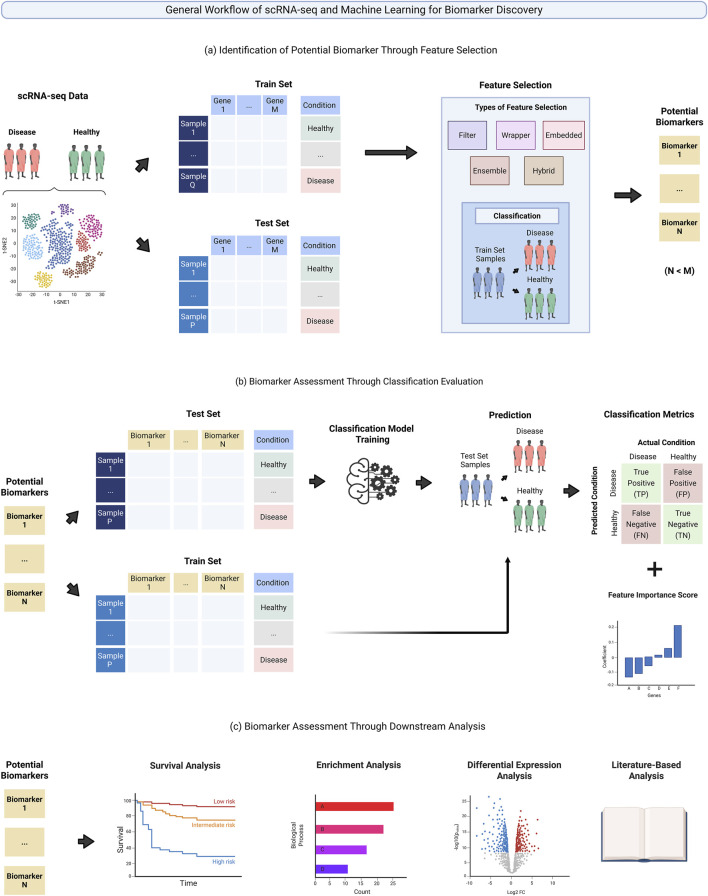
General workflow of scRNA-seq and machine learning for biomarker discovery. **(a)** Single cell sequencing data comprised of healthy and disease samples is first split into training and test sets. The training set is utilized as input for feature selection to identify potential biomarkers. Identified biomarkers then undergo further evaluation through two approaches: **(b)** The assessment of classification performance through classification metrics and feature importance score, and **(c)** The assessment of biological relevance through downstream analysis.

The application of machine learning and scRNA-seq for biomarker discovery typically follows a general workflow involving feature selection to identify potential biomarkers, followed by classification and downstream evaluation to assess their significance. To begin, the initial scRNA-seq data undergoes standard single-cell preprocessing analysis, including quality control, normalization, batch correction, clustering, and cell type annotation (Hayakawa and Ozaki, 2025). Once preprocessing is complete, the dataset is partitioned into training and test sets. This partitioning is typically performed on the normalized gene expression matrix (Figure 1a), although the exact strategy may vary depending on the specific analysis framework (e.g., cell-level vs. patient level analysis) (Section 3). Feature selection is then performed using only the training set to identify genes that are predictive of specific diseases or conditions, thereby reducing the full set of genes to a smaller subset of potential biomarkers (Figure 1a). Ideally, the selected features, i.e., biomarker candidates, should demonstrate both strong predictive performance and biological relevance to the disease or condition of interest. Therefore, further evaluation is necessary to validate their classification performance (Figure 1b) and biological significance (Figure 1c).

To evaluate the biomarker’s predictive capability, genes selected by feature selection are used to develop a classification model, which is first trained on the training set and then evaluated on the test set (Figure 1b). While many workflows generally rely solely on a training and test set for model evaluation, some frameworks additionally incorporate a third independent set, termed as a validation set, to tune hyperparameters and perform cross-validation to improve model generalizability and reduce the risk of overfitting (Zhou et al., 2024; Kang et al., 2022; Xu and Goodacre, 2018). The classification performance of the potential biomarkers is then evaluated using various classification metrics and feature importance scores. Subsequently, to assess for biological relevance, potential biomarkers also undergo a series of downstream biological analysis. These include examining their association with patient survival outcomes, their involvement in disease-related pathways and biological processes, their differential expression patterns, and existing literature evidence supporting their relevance to the disease of interest (Figure 1c).

Although most machine learning approaches for scRNA-seq biomarker discovery follows this general workflow (Figure 1), the specific choice of method that is applied for each major aspects of the framework often differ (Table 1). These variations are further explored in this paper, organized according to differences in 1) biomarker discovery level (Section 3), 2) supervised learning algorithms (Section 4.1), 3) feature selection methods (Section 4.2), 4) classification metrics (Section 5.1), and 5) downstream analysis evaluations (Section 5.2).

**TABLE 1:** Overview of current scRNA-seq and machine learning for biomarker discovery studies, highlighting key differences in disease application, biomarker discovery level, machine learning algorithms, feature selection method, classification metrics and downstream analysis.

Disease	scRNA-seq reference	Biomarker discovery method	Machine learning algorithms	Feature selection method	Classification metrics	Downstream analysis
Type 1 Diabetes	Patil et al. (2024)	Cell-level method	XGBoost support vector machine random Forest	Embedded	Accuracy	Literature-based validation go and kegg enrichment analysis
Hepatocellular carcinoma	Li H. et al. (2022)	Cell-level method	Support vector machine	Hybrid	Matthew’s correlation coefficient	Literature-based validation go enrichment analysis
Alzheimer’s disease	Krokidis et al. (2023a)	Cell-level method	Random forest	Ensemble	Accuracy	Literature-based validation go enrichment analysis
COVID-19	Li W. et al. (2022)	Cell-level method	Decision trees random forest	Hybrid	Precision Recall F1-score	Literature-based validation go and kegg enrichment analysis
Preeclampsia	Zhou et al. (2024)	Cell-level method	Random forest	Ensemble	AUROC	Literature-based validation
Melanoma	Kang et al. (2022)	Patient-level method	Random forest xgboost neural network	Wrapper	Accuracy	Survival analysis gene set enrichment analysis differential expression analysis
Melanoma	Dong et al. (2024)	Patient-level method	Logistic regression random forest support vector machine naïve bayes	Embedded	AUROC	Literature-based validation differential expression analysis
Skin cancer and pancreatic ductal carcinoma	Liu et al. (2022)	Cell-level method	Random forest support vector machine adaboost decision trees	Filter	Accuracy AUROC	Literature-based validation differential expression analysis
Prostate cancer	Zhang et al. (2024)	Patient-level method	XGBoost support vector machine random forest	Hybrid	AUROC	Literature-based validation gene set enrichment analysis go enrichment analysis
COVID-19	Cao et al. (2023)	Patient-level method	Random forest support vector machine xgboost neural network	Embedded	Precision recall F1-score	Literature-based validation
Heart Failure	Yuan et al. (2024)	Cell-level method	Logistic regression support vector machine naïve bayes	Embedded	AUROC	Literature-based validation gene set enrichment analysis kegg enrichment analysis
COVID-19	Goel et al. (2022)	Patient-level method	Random forest support vector machine catBoost	Embedded	AUROC	Literature-based validation

## 3 Cell-level vs. patient-level methods for scRNA-seq biomarker discovery

**FIGURE 2:**
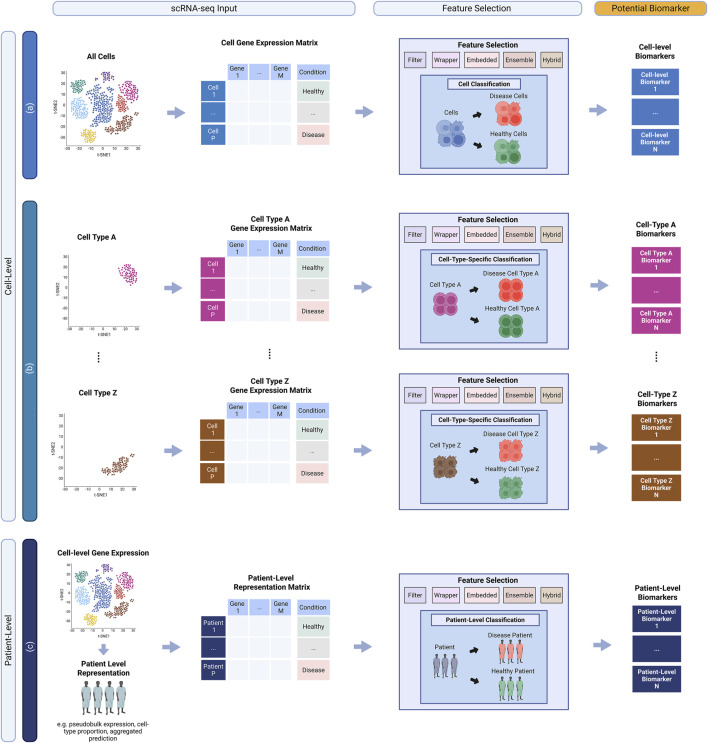
Overview of different classification methods for scRNA-seq machine learning biomarker discovery. **(a)** General Cell Method: a framework where all cells are used for disease prediction and identification of potential biomarkers. **(b)** Cell-type-specific Method: a framework where specific cell-type groups are used for disease prediction and identification of potential biomarkers. **(c)** Patient-level Method: a framework where cell-level gene expression is first transformed to patient-level representation to then identify of potential biomarkers.

In scRNA-seq, the application of machine learning for biomarker discovery can be differentiated by the “level” at which the methods are applied. Traditionally, biomarker identification through machine learning methods focuses on classification performance in predicting individual patients (patient-level methods). However, since scRNA-seq captures gene expression at the individual cell level, it also enables the capability to identify potential biomarkers through the classification of individual cells (cell-level method). As a result, current applications of machine learning for scRNA-seq biomarker discovery can be divided into two distinct categories: 1) cell-level methods (Figures 2a,b) and 2) patient-level methods (Figure 2c).

### 3.1 Cell-level methods

Biomarker discovery via cell-level methods focuses on using individual cells, rather than patients, to identify predictive genes (Figures 2a,b). By applying feature selection and supervised learning to cell-level gene expression data, these methods aim to classify cells by disease or condition, enabling biomarker identification within and across cell populations. As there are significantly more cells than there are number of patients, cell-level methods can overcome issues of dataset size limitation. By increasing the number of instances a machine learning model can train on, it maximises the likelihood of improved classification performance and the identification of more robust biomarkers (Patil et al., 2024).

Current application of cell-level classification methods for scRNA-seq biomarker discovery can be further divided into two distinct approaches: 1) General cell methods (Figure 2a) and 2) Cell-type-specific methods (Figure 2b).

#### 3.1.1 General cell methods

The main principle behind the general cell method is to identify biomarkers that can distinguish between disease or condition groups across different cell type populations. This approach assumes that all cells are treated equally during feature selection and classification, regardless of their specific cell type, focusing only on the cell’s disease condition label. If we assume that relevant biomarkers are consistently present across all cell types, then the larger number of cells available for training will likely enable general cell method to achieve better performance compared to approaches that use only specific cell types, which are limited by smaller dataset sizes.

The application of general cell methods for biomarker discovery has been utilized across various diseases. Li et al. identified potential diagnostic biomarkers of hepatocellular carcinoma (HCC) by training a machine learning model to accurately differentiate between HCC cells from normal liver cells (Li W. et al., 2022). Similarly, in their analysis of Alzheimer’s disease (AD), Krokidis et al. developed a general cell classification model that identified key differences between cells originating from AD patients and those from healthy controls (Krokidis et al., 2023a). Furthermore, Patil et al. expanded on the general binary framework, by applying machine learning classification to predict cells from three different condition groups (non-diabetic control, non-diabetic but islet AAb-positive (AAb+), and type 1 diabetes), revealing distinct gene signatures associated with type 1 diabetes progression (Patil et al., 2024).

#### 3.1.2 Cell-type-specific methods

In contrast to general cell methods, cell-type-specific methods aim to identify potential biomarkers by applying feature selection and supervised learning classification within specific cell-type populations (Figure 2b). The main motivation behind this approach is to harness scRNA-seq’s ability to explore cell heterogeneity by facilitating the identification of cell-type specific biomarkers, which is not possible when using the general cell method. Additionally, as each machine learning model is trained on a specific cell type, comparing the predictive performance across cell-type specific models can help identify cell types that are most predictive for the disease. However, a key limitation of this approach is that some cell-type populations may contain very few cells, which can result in insufficient data to train a reliable model and hinder the discovery of robust cell-type-specific biomarkers.

Cell-type-level methods for biomarker discovery have been applied to a wide range of diseases. Li et al. identified COVID-19-specific immune markers within multiple different cell-types (Li H. et al., 2022). In their analysis, they define classification rules to distinguish cells from healthy, COVID-19, acute systemic inflammatory response (lipopolysaccharide), and non-COVID-19 severe respiratory disease samples across four different cell types (B cells, CD4^+^ T cells, CD8^+^ T cells, and natural killer cells), revealing cell-type specific COVID-19-related biomarkers and highlighting classification differences between cell types (Li H. et al., 2022). Similarly, Zhou et al. applied cell-type-specific classification methods within monocytes, CD4^+^ naïve T cells, CD8^+^ naïve T cells, and Treg cell-type groups to identify cell-type specific biomarkers capable of distinguishing cells from individuals with normal pregnancies from those with preeclampsia (Zhou et al., 2024).

### 3.2 Patient-level methods

In contrast to cell-level methods, biomarker discovery through a patient-level method revolves around the use of individual patients as the primary subject of discovery (Figure 2c). The overall goal is to utilize feature selection and supervised learning to identify highly predictive genes that can be used by a model to classify individual patients into their respective disease or condition group of interest. However, as scRNA-seq captures gene expression at the individual cell level, rather than at the patient level, patient-level methods often require an additional preprocessing step that facilitates patient prediction from cell-level gene expression data.

One approach to facilitate patient-level methods is to first transform cell-level gene expression into patient-level gene expression. This can be achieved through the generation of a pseudo-bulk gene expression, where the mean expression values of each gene are aggregated across all cells for each patient. The transformed pseudo-bulk gene expression can then be used as input to train the classification model. For example, Kang et al. applied this approach to predict patient responses to immune checkpoint blockade therapy (responder vs. non-responder), identifying potential biomarkers associated with melanoma treatment outcomes (Kang et al., 2022).

Alternatively, biomarker discovery through patient-level methods can also be achieved by aggregating the prediction probability of cell-level classification models. In their development of scPANEL, Xie et al. achieved patient-level prediction by aggregating the area-under-curve (AUC) score of cell-level prediction and adjusting the classifiers prediction weights based of the number of cells in each patient (Xie et al., 2024).

In addition to gene-based features as input, the proportion of cell types in each patient can also be used. This can be obtained first through annotation of single-cell data sets to identify cell types for each patient (Phipson et al., 2022). To predict the immunotherapy response of melanoma patients, Dong et al. used patient cell-type proportions to train a classification model that distinguishes between responders and non-responders to immune checkpoint inhibitor therapy (Dong et al., 2024). Their analysis identified key predictive cell-type and cell-type proportion biomarkers that are significant for predicting immunotherapy responses in melanoma patients.

Other patient-level methods for machine-learning biomarker discovery includes diverse examples such as ProtoCell4P (Xiong et al., 2023), CloudPred (He et al., 2022), and scRat (Mao et al., 2024). Unlike cell-level methods, which can generally be formulated as disease classification using cell-by-gene expression matrices, patient-level approaches must address the challenge of utilizing cell-level information for patient-level analysis. Consequently, existing patient-level methods are typically more methodologically diverse, employing distinct modeling strategies tailored to this aggregation problem.

## 4 Supervised learning algorithms and feature selection methods for scRNA-seq biomarker discovery

### 4.1 Supervised learning algorithms for biomarker discovery

The application of machine learning for scRNA-seq biomarker discovery encompass various supervised learning algorithms, which differ in how they assess and rank features for classification. As a result, the selection of potential biomarkers can vary widely depending on the algorithm employed (Zhang et al., 2021). In this section we explain the fundamental concepts of how different supervised learning algorithms can be applied for scRNA-seq biomarker discovery. We chose papers that covers the application of the algorithm or method across a wide range of variations within scRNA-seq biomarker discovery, such as different disease, biomarker discovery method, feature selection method, with details in Table 1.

#### 4.1.1 Decision trees

Decision trees (DTs) is a supervised learning algorithm that models’ decisions and their possible consequences through a tree-like structure (Rokach et al., 2005). DTs can be harnessed for biomarker discovery by representing each internal node of the tree as a specific input feature (e.g., gene), while the leaves represent the predicted outcome (e.g., disease condition). Potential biomarker can be identified by evaluating each gene’s importance within a tree, selecting genes that maximize class separation at each decision point through the calculation of splitting criteria metrics (e.g., Impurity, Information-gain, Gini Index) (Rokach et al., 2005). DTs have been applied for scRNA-seq biomarker discovery by Patil et al. (2024), Li H. et al. (2022) and Liu et al. (2022).

#### 4.1.2 Random forest

Random forests (RF) is a supervised learning algorithm that builds on the concept of decision trees (DTs) by aggregating the predictions of multiple DTs to improve accuracy and robustness (Breiman, 2001). For biomarker discovery, similarly to DTs, RF represents an input feature (genes) as an internal node and the output outcome (disease condition) as a leaf. However, as RF incorporate multiple DTs, each tree is trained on a different subset of the input data, with the outcome of each tree aggregated into a single final prediction. Potential biomarkers can be identified by evaluating the importance of a gene across trees, using feature importance scores such as (e.g., Gini importance, permutation based variable importance (Qi et al., 2012). Both Zhang et al. (2024) and Ma et al. (2022) have demonstrated the use of RF for scRNA-seq biomarker discovery.

#### 4.1.3 Boosting algorithms

Boosting algorithms (e.g., XGBoost, AdaBoost, etc.) are supervised learning methods that utilize an ensemble of weak learners (typically decision trees) (Schapire, 2013; Chen and Guestrin, 2016). However, unlike Random Forest, which follows a bagging approach, boosting algorithms build trees sequentially, with each new tree correcting the errors of the previous ones (Ferreira et al., 2012). Boosting algorithms have been widely applied for biomarker discovery using scRNA-seq data (Dong et al., 2024; Patil et al., 2024; Liu et al., 2022; Cao et al., 2023; Peng et al., 2005). Boosting algorithms is utilized for biomarker discovery by identifying genes that contribute to reducing misclassification across learning iterations. Genes that consistently improve model performance are considered more informative and have greater potential as biomarkers.

#### 4.1.4 Logistic regression

Logistic regression (LR) is a supervised learning algorithm that uses a logistic function to predict the likelihood of an outcome (ranging from 0 to 1) based on a given input (Nick et al., 2007). LR can be applied for biomarker discovery by fitting a logistic function to model the relationship between input features (genes) and the probability of a specific outcome (e.g., disease condition). Potential biomarkers are identified by calculating the coefficient for each gene, where the coefficient represents the change in the log-odds of the outcome. Larger coefficients indicate a stronger input-output association (Saarela and Jauhiainen, 2021) and greater potential as biomarkers. LR have been applied by Dong et al. (2024), Krokidis et al. (2023b) and Yuan et al. (2024) for scRNA-seq biomarker discovery.

#### 4.1.5 Support vector machines

Support vector machines (SVM) is a supervised learning algorithm that aims to find the optimal hyperplane that separates data points of different classes (Suthaharan and Suthaharan, 2016). In the context of biomarker discovery, SVM can be applied by mapping input features (e.g., gene expressions) into a higher-dimensional space using a kernel function, where the algorithm then identifies the hyperplane that best separates the different classes (disease conditions). Potential biomarkers can be identified by examining each genes weight or coefficient within the support vector’s decision function. Genes with higher weights/coefficients indicates its influence in class separation and classification, serving as indicator of potential biomarkers. Patil et al. (2024), Li W. et al. (2022), Dong et al. (2024) and Cao et al. (2023) all applied SVM for scRNA-seq biomarker discovery.

#### 4.1.6 Naïve Bayes

Naïve Bayes (NB) is a supervised learning algorithm based on Bayes’ theorem, which assumes that features are conditionally independent given the class labels (Webb et al., 2010). For biomarker discovery, Naïve Bayes can be applied by estimating the conditional probabilities of an input feature (gene) given a specific outcome (disease condition). Potential biomarkers are identified by analysing each genes contribution to the likelihood of an outcome, with features with significant contribution having greater potential as biomarkers candidate. Naïve Bayes has been applied by both Patil et al. (2024) and Dong et al. (2024) for scRNA-seq biomarker discovery.

#### 4.1.7 Neural network algorithms

Neural network algorithms draw inspiration from the structure of the human brain and utilise layers of neurons for machine learning. When applied for deep learning (neural network with more than three neural layers) neural networks can learn complex patterns from large amount of data. Neural networks encompass a wide range of distinct variations including feedforward neural networks (FFNN), generative adversarial networks (GANs), autoencoders (AE) and many others that differ in their overall architecture and function (Sarker, 2021b). It is also important to note that neural networks typically require more data instances to train on compared with other machine-learning algorithms. Therefore, the choice between using a neural network-based algorithm for biomarkers discovery over other machine learning algorithms often depends on the size of the dataset available.

A common neural network utilized for scRNA-seq biomarker discovery is FFNN (Kang et al., 2022; Wang et al., 2023). FFNN is a type of neural network where information flows in one direction, from the input layer through one or more hidden layers, and finishes at the output layer. For biomarker discovery, FFNN is typically structured with an input layer consisting of features (genes) and an output layer representing the outcome (e.g., disease condition). The hidden layers between the input and output enable the network to learn the relationship between input and output by adjusting the weights of the neurons (Sarker, 2021b). Potential biomarkers are identified by analysing how much each gene contributes to the predictive performance of the neural network (Kang et al., 2022).

### 4.2 Feature selection for biomarker discovery

**FIGURE 3:**
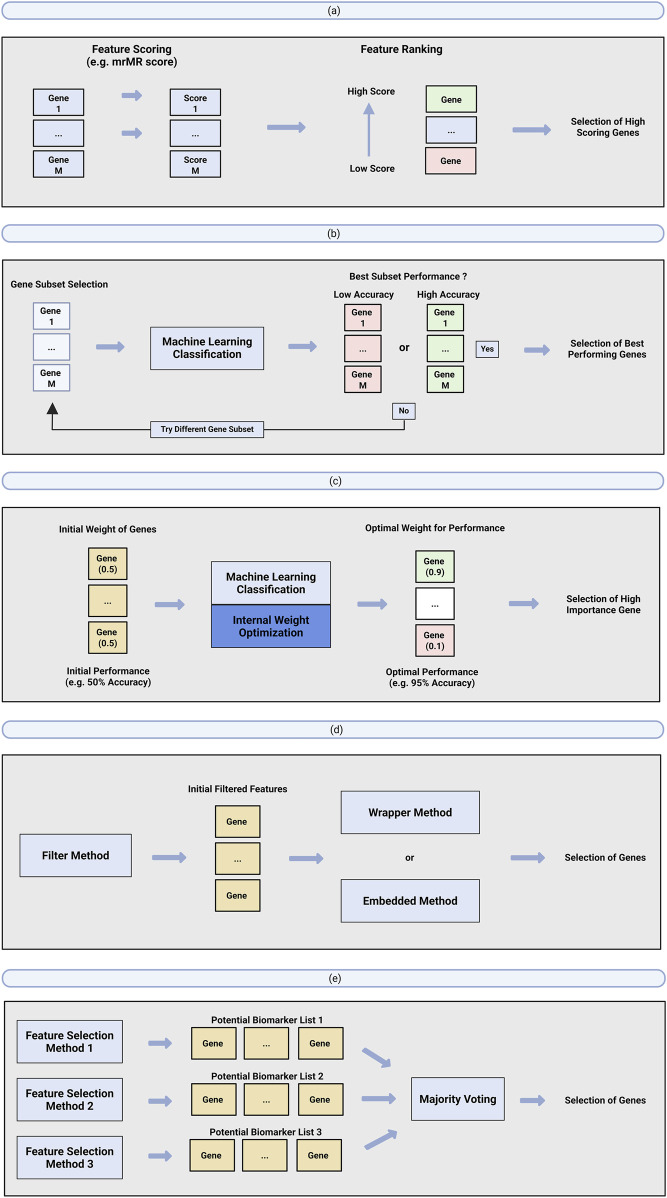
Overview of different feature selection methods that have been applied for scRNA-seq machine learning biomarker discovery. **(a)** Filter method, **(b)** wrapper method, **(c)** embedded method, **(d)** hybrid method and **(e)** ensemble method.

In machine learning, feature selection aims to identify the most relevant features from a dataset to improve model performance, reduce overfitting, and aid interpretability by removing uninformative or irrelevant features (Pudjihartono et al., 2022). Subsequently, feature selection can be harnessed for biomarker discovery by selecting genes that are significantly relevant for predicting different disease conditions or outcome groups (Zhang et al., 2021). Feature selection can be applied either prior to or during classification, with various strategies employed to identify the most relevant features. These feature selection methods are typically categorized into 1) filter methods, 2) wrapper methods, 3) embedded methods, 4) hybrid methods and 5) ensemble methods (Figure 3).

#### 4.2.1 Filter methods

Filter methods is a type of feature selection that identifies important features prior to and independently of the classification model, typically by ranking them based on traditional statistical tests and metrics (Figure 3a). As a result, filter methods are categorized as a traditional statistical approach and thus do not benefit from the advantages of machine learning algorithms, such as the consideration of feature interaction during selection (Pudjihartono et al., 2022). Nevertheless, they remain valuable for biomarker discovery and are commonly implemented using algorithms such as minimal-redundancy-maximal-relevance (mRMR) algorithm (Peng et al., 2005), mutual information (MI) (Battiti, 1994), analysis of variance (ANOVA) (St and Wold, 1989) and many others. More importantly, filter methods play a crucial role in machine learning solutions for biomarker discovery, as filter methods are often used in conjunction with other feature selection methods (wrapper or embedded) as part of a hybrid approach (Section 3.3.4).

#### 4.2.2 Wrapper methods

Wrapper methods identify potential biomarkers based on the machine learning model’s performance (Figure 3B). Wrapper methods approach the biomarker discovery process as an iterative search problem, evaluating the classification performance of different combination of genes, and selecting the best performing gene set as potential biomarkers. The feature selection approach is termed ‘wrapper’ as the iterative evaluation to determine potential biomarkers is wrapped around the machine learning model’s performance.

The application of wrapper methods in scRNA-seq biomarker discovery has been explored through the use of recursive feature elimination (RFE) (Kang et al., 2022; Zhang et al., 2022). RFE functions by initially using all genes for classification and ranking them based on their importance or contribution to the model. It then removes the least important feature and recalculates the prediction accuracy of the new set. This process is continued and iterated upon until a desired accuracy performance or number of features is reached, with the final set of genes representing potential biomarkers. Similarly, other wrapper methods applied in scRNA-seq biomarker discovery include the use of Boruta (Zhou et al., 2024; Li H. et al., 2022), a random forest-based wrapper that iteratively evaluates feature importance by comparing actual features with randomly permuted shadow features (Kursa et al., 2010), and incremental feature selection (IFS) (Li H. et al., 2022; Huang et al., 2021), which iteratively adds and evaluates features based on their impact on model accuracy (Liu and Setiono, 1998).

As wrapper methods evaluates the predictive capabilities of gene subsets (not individual genes) through machine learning performance, the identification of potential biomarkers benefits from the consideration of feature interaction (Pudjihartono et al., 2022). However, due to the iterative nature of wrapper methods, they can be computationally demanding (Chandrashekar and Sahin, 2014).

#### 4.2.3 Embedded methods

Similar to wrapper methods, embedded methods represent a biomarker discovery strategy that selects potential biomarkers based on a gene’s importance or contribution to the performance of a machine learning model. However, unlike wrapper methods, which iteratively evaluate different gene combinations to find potential biomarkers, embedded methods integrate the biomarker identification process directly into the machine learning model training (Figure 3c). This approach optimizes classification performance by adjusting the importance score of each gene, identifying the optimal function weights that maximize accuracy, and selecting genes with the highest significance or contribution to the model’s predictions as potential biomarkers.

The use of embedded methods for scRNA-seq biomarker discovery is exemplified by the application of the Least Absolute Shrinkage and Selection Operator (LASSO) algorithm (Cao et al., 2023; Wang et al., 2023). LASSO functions by applying an L1 penalty to the coefficients of genes during model training. This penalty encourages sparsity by shrinking coefficients based on their contribution to the model’s performance, so that as the model trains, genes with less importance have their coefficients reduced to zero, effectively excluding them from the final model (Tibshirani, 1996). This process continues until a specified number of features, or a desired performance metric is achieved, resulting in a final set of genes of high predictive importance, which serve as potential biomarkers. Other biomarker discovery efforts using embedded methods revolve around the assessment of feature importance scores of different genes through tree-based algorithms (Dong et al., 2024; Patil et al., 2024; Goel et al., 2022).

Embedded feature selection methods provide a unique balance between efficiency and predictive performance for biomarker discovery. By integrating feature selection directly into the model training process, these methods inherently account for feature interaction during the evaluation of predictive features. Additionally, embedded methods are generally more computationally efficient compared to wrapper methods, as they integrate feature selection directly into the training process of the model, avoiding the need for an exhaustive search over all possible feature subsets (Pudjihartono et al., 2022). However, embedded methods are not without limitations. Their reliance on the specific machine learning algorithm being employed means that the feature selection process is inherently tied to that algorithm’s assumptions and biases (Pudjihartono et al., 2022).

#### 4.2.4 Hybrid methods

Hybrid methods is a feature selection approach that integrate multiple feature selection methods into a multi-step process, leveraging the strengths of each individual method to enhance overall performance (Figure 3d). Typically, hybrid methods begin with an initial filter method, followed by either a wrapper or embedded method. Filter methods are known for their ability to reduce the search space efficiently, while wrapper/embedded methods, which takes into consideration feature interactions and model dependencies, generally result in better model performance (Pudjihartono et al., 2022). Thus, the structure of the hybrid approach, where the filter method precedes the wrapper or embedded method, enables feature selection to maximize both performance and computational efficiency. However, since the initial search space reduction is done through traditional statistical filters, hybrid methods may suffer from the same limitation as traditional biomarker discovery tools. Thus, the application of hybrid methods for biomarker discovery represents a middle-ground approach between traditional statistics and machine learning.

A common approach for hybrid methods in scRNA-seq biomarker discovery is the application of DGE analysis as an initial filter method, followed by the identification of highly predictive genes through wrapper/embedded methods (Ma et al., 2022; Yuan et al., 2024; Sultana et al., 2023). The use of statistical tests to identify differentially expressed genes offers a straightforward yet well-established approach for selecting potential biomarkers, which can then be further assessed for predictive capability using machine learning models. Moreover, evaluating genes as potential biomarkers through both traditional statistical methods and machine learning enhances the robustness of the biomarkers. Other hybrid approaches include the initial application of the minimal-redundancy-maximal-relevance (mRMR) algorithm (Li W. et al., 2022; Li H. et al., 2022) or Seurat’s built-in variance stabilizing transformation (VST) function (Liu et al., 2022) as a filter, followed by an embedded or wrapper method.

#### 4.2.5 Ensemble methods

Similarly to hybrid methods, ensemble methods is an approach that combine two or more different feature selection methods (Figure 3e). However instead of a sequential approach applied in hybrid methods, ensemble method applies a parallel consensus approach. Ensemble hybrid methods function by initially identifying feature subsets of different feature selection methods separately. Then, a consensus-voting framework is applied to identify overlapping features that are selected by multiple independent methods. The main motivation behind an ensemble approach is to identify potential biomarkers through the assessment of robustness and stability. By focusing only on overlapping features, ensemble methods filter out genes that may be highly predictive in one method but not generalized across different approaches.

The application of ensemble method can be applied through any combination of filters, wrapper or embedded methods. In their exploration of Alzheimer’s disease using scRNA-seq data of the hippocampus and cortex region, Krokidis et al. (2023a) utilized an ensemble feature selection method by combining a filter method (differential expression analysis) and an embedded method (XGBoost feature importance score). Additionally, in a separate biomarker discovery study on Alzheimer’s disease through peripheral blood scRNA-seq data, Krokidis et al. (2023b) developed another ensemble feature selection method that combined three machine learning feature selection algorithms (logistic regression, triku and random-forest). To identify overlapping features across methods, both studies utilized a Borda consensus voting. Borda voting is a ranked voting method where each features receives points based on their position in each feature selection method ranking, with higher-ranked choices getting more points, and the feature with the highest total points being selected as potential biomarkers.

## 5 Classification and downstream biological assessment of potential biomarkers

Ideally, biomarkers identified through feature selection should not only enable accurate prediction of conditions but also hold meaningful biological associations with the underlying disease of interest. To ensure the reliability and utility of these candidate biomarkers, it is essential to conduct further comprehensive evaluation that assess both their predictive effectiveness in classification tasks (Section 5.1) and their overall relevance as potential biomarkers (Section 5.2). This dual-layered assessment ensures that the selected genes are not only statistically significant but also biologically meaningful, strengthening their potential as genuine biomarkers.

### 5.1 Assessment through classification performance evaluation

**FIGURE 4:**
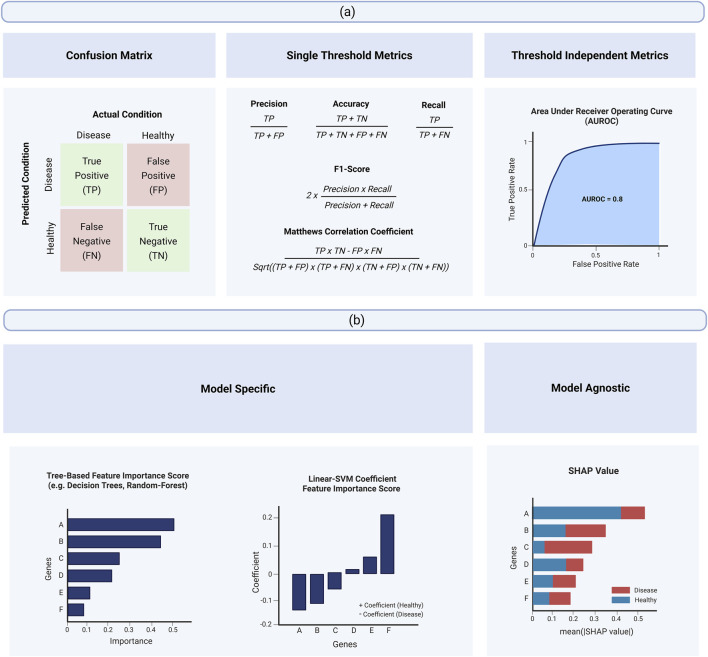
Overview of assessment of classification performance through the application of classification metrics and feature importance score. **(a)** Variation of classification metrics. **(b)** Variation of feature importance score.

The evaluation of classification performance of selected biomarkers is typically measured using two main metric types: 1) classification metrics (Figure 4a) and 2) feature importance scores (Figure 4b).

Classification metrics evaluate how effectively the selected biomarkers can predict different disease conditions in the new data (test set). However, the quantification of predictive performance only reflects the classification capability of the entire set of potential biomarkers and does not provide insight into the individual importance of each gene. To understand the significance of each biomarker, feature importance scores must be derived from the classification model. Feature importance score quantifies how much each feature contributes to a model’s decision-making process and predictions (Saarela and Jauhiainen, 2021). For biomarker discovery, feature importance score helps explain which biomarkers are more important and indicate whether they are predictive of healthy or disease condition. When used together, classification metrics and feature importance score provide comprehensive evaluation of the predictive capability of potential biomarkers, with classification metrics providing a measure of strength and feature importance scores providing explainability.

#### 5.1.1 Classification metrics for biomarker discovery

##### 5.1.1.1 Prediction outcome and confusion matrix

The evaluation of a classification model’s performance and its selected genes is based on the four possible prediction outcomes: (FDA-NIH Biomarker Working Group, 2016): True Positive (TP)—correctly predicting a disease sample as diseased; (Vargas and Harris, 2016); True Negative (TN)—correctly predicting a healthy sample as healthy; (Kraus, 2018); False Positive (FP)—incorrectly predicting a healthy sample as diseased; and (Kim et al., 2021) False Negative (FN)—incorrectly predicting a disease sample as healthy. These four outcomes are typically summarized in a confusion matrix, which provides a structured view of a model’s performance. More importantly, these four possible classification outcomes serve as the building blocks for the other classification metrics (Rainio et al., 2024) (Table 2). Additionally, since different metrics evaluate distinct aspects of classification performance and no single metric is universally accepted as the best, it is generally recommended to use multiple metrics for a comprehensive assessment (Miller et al., 2024).

**TABLE 2:** Overview of various classification metrics that can be used to evaluation potential biomarkers, highlighting key difference in formula, advantages and disadvantages.

Metric	Description	Formula	Pros	Cons	Application in scRNA-seq
Accuracy	The proportion of all predictions (both positive and negative) that are correct	$\frac{TP+TN}{TP+TN+FP+FN}$	Simple and clear to interpret	Heavily impacted by imbalanced datasets (Rainio et al., 2024; Miller et al., 2024)	Patil et al. (2024) Cao et al. (2023) Wang et al. (2023)
Precision	The proportion of predicted positives that are positive	$\frac{TP}{TP+FP}$	Valuable in scenarios where false positives are more detrimental than false negatives (Rainio et al., 2024)	Does not account for false negatives	Wang et al. (2023)
Recall	The proportion of actual positives that are correctly identified	$\frac{TP}{TP+FN}$	Useful when false negatives are more detrimental than false positives (Miller et al., 2024)	Does not account for false positives	Wang et al. (2023)
F1-Score	The harmonic means of precision and recall	$2\times \frac{Precision\times Recall}{Precision+Recall}$	Suitable for Imbalanced Dataset (Miller et al., 2024; Lundberg and Lee, 2017)	Does not account for true negatives	Li W. et al. (2022) Li W. et al. (2022) Cao et al. (2023)
Matthews Correlation Coefficient (MCC)	A balanced measure of classification quality that accounts for true and false positives and negatives	$\frac{TP\times TN-FP\times FN}{\surd \left(TP+FP\right)\times \left(TP+FN\right)\times \left(TN+FP\right)\times \left(TN+FN\right)}$	Suitable for Imbalanced Dataset (Miller et al., 2024; Lundberg and Lee, 2017)	MCC values range from −1 to +1, which can be less intuitive to interpret compared to other metrics which generally values range from 0 to 1	Li H. et al. (2022) Li W. et al. (2022)
Area Under Receiver Operating Curve (AUROC)	A threshold-independent metric representing the model’s ability to distinguish between positive and negative classes across all possible classification thresholds	$False\;Positive\;Rate\;\left(FPR\right)=\frac{FP}{FP+TN}$ $True\;Positive\;Rate\;\left(TPR\right)=\frac{TP}{TP+FN}$ $\text{AUROC}=\int_{0}^{1}\text{TPR}\left(\text{FPR}\right)\;d\left(\text{FPR}\right)$	Provides evaluation of performance across all possible classification threshold	Susceptible to biases from imbalanced datasets (Miller et al., 2024)	Dong et al. (2024) Liu et al. (2022) Yuan et al. (2024)

#### 5.1.2 Feature importance scores

Feature importance scores quantify how much each feature contributes to a model’s decision making process and predictions, helping to identify which features are most influential in determining accurate classification (Saarela and Jauhiainen, 2021). For scRNA-seq biomarker discovery, feature importance scores can serve two purposes: 1) to identify potential biomarkers during feature selection through wrapper or embedded methods (Section 4.2.2 and 4.2.3) (Dong et al., 2024; Patil et al., 2024; Yuan et al., 2024) or 2) be utilized to interpret the significance of each individual biomarkers (Zhou et al., 2024; Kang et al., 2022; Ma et al., 2022; St and Wold, 1989; Goel et al., 2022). There are two types of feature importance scores in scRNA-seq biomarker discovery (Figure 4b): 1) model-specific (Kang et al., 2022; Ma et al., 2022) and 2) model-agnostic feature importance scores (Zhou et al., 2024; Goel et al., 2022).

Model-specific feature importance scores (Figure 4b) are derived directly from the internal parameters or structure of a specific machine learning algorithm. As each supervised learning algorithm utilizes different learning approaches for classification, these feature importance score reflects different contribution and does not generalize across different algorithms. For example, tree-based models like decision trees, random-forest and XGBoost measures feature importance by assessing how much each feature reduces impurity when used to split nodes (Qi et al., 2012). In contrast, logistic regression evaluates feature importance based on the magnitude of feature coefficients (Nick et al., 2007), while support vector machines (linear kernel) determine feature importance by examining the weight of each feature in the decision boundary (Suthaharan and Suthaharan, 2016). As a result, the application of a model-specific feature importance score to interpret the classification performance of potential biomarkers can only be done within the context of the same algorithm and should not be used for direct comparison across different models.

In contrast, model-agnostic feature importance scores are designed to evaluate the contribution of individual features independently of the underlying machine learning algorithm (Figure 4b). Among model-agnostic scores, SHAP (Shapley Additive Explanations) has emerged as a widely adopted approach. SHAP is based on cooperative game theory and quantifies the contribution of each feature by considering all possible features (Lundberg and Lee, 2017). Unlike model-specific methods, SHAP can be applied consistently across different classifiers, making it especially valuable for comparing feature importance across models. This property is particularly advantageous in biomarker discovery, as SHAP values provide consistent and interpretable estimates of each gene’s contribution regardless of the algorithm used. Such consistency facilitates the accumulation of robust evidence for candidate biomarkers across diverse algorithmic methods.

### 5.2 Assessment through downstream analysis evaluation

Once the selected genes have been evaluated for their classification performance, their overall relevance towards the disease of interest must be further assessed before they can be considered as potential biomarkers (Figure 1c). This evaluation typically involves a range of downstream biological analyses, differential gene expression validation and existing literature verification aimed at validating whether the potential biomarkers are relevant and associated with the disease or condition of interest.

#### 5.2.1 Validation of biological relevance

##### 5.2.1.1 Enrichment analysis

Enrichment analysis is a key downstream approach used to evaluate the biological relevance of machine learning selected biomarkers by determining whether they are significantly associated with known biological processes, functions, or pathways relevant within a specific disease. This analysis is typically conducted using either Gene Set Enrichment Analysis (GSEA) or Over-Representation Analysis (ORA), applied to curated databases such as gene ontology (GO), Kyoto encyclopedia of genes and genome (KEGG), or the molecular signature database (MSigDB) (Ashburner et al., 2000; Kanehisa and Goto, 2000; Subrama et al., 2005; Liberzon et al., 2011). For example, by performing enrichment analysis using GO and KEGG database, Huang et al. (2021) and Sultana et al. (2023) were able to provide biological insights into whether the identified biomarkers are significantly associated with specific biological processes, molecular functions, or pathways known to be linked to the disease or condition of interest. Similarly, Kang et al. (2022) and Yuan et al. (2024) utilized GSEA to evaluate whether their machine learning selected biomarkers where biologically relevant to their disease or condition of interest.

##### 5.2.1.2 Survival analysis

Survival analysis is another key downstream approach used to evaluate the clinical relevance of selected genes by determining their association with time-to-event outcomes, such as overall survival, disease-free survival, or progression-free survival. Commonly used survival analysis techniques include the Kaplan-Meier estimator, which provides a non-parametric estimate of the survival function, and the Cox proportional hazards model, which assesses the relationship between biomarker expression levels and the hazard of experiencing a clinical event (Kang et al., 2022). These methods have been widely adopted in scRNA-seq studies to validate potential biomarkers by providing insight into whether selected genes have prognostic value in clinical settings (Kang et al., 2022; Sultana et al., 2023).

#### 5.2.2 Validation through differential gene expression analysis

A hallmark of robust biomarkers is their consistent identification across different feature selection methods. To assess this robustness, it is common to apply a secondary feature selection method as a validation step. In the context of the application of machine learning for scRNA-seq biomarker discovery, DGE analysis is frequently used for this secondary validation (Kang et al., 2022; Dong et al., 2024; Liu et al., 2022).

Differential gene expression analysis is a statistical method used to identify genes that show significant changes in expression levels between different biological conditions or disease groups. For machine learning biomarker discovery, DGE analysis can serve two purposes: 1) be utilized for feature selection as a filter method or a part of hybrid method (Section 4.1and 4.4) or 2) be utilized as a downstream analysis approach that helps validate the biological relevance of potential biomarkers. When used to help assess the biological relevance of potential biomarkers, DGE analysis provides an additional layer of evidence that supports the potential of selected genes as meaningful biomarkers. Importantly, integrating DGE analysis into machine learning–based biomarker discovery enables comparison between machine learning selected genes and those reported in conventional, non-machine learning studies (Kang et al., 2022; Dong et al., 2024; Liu et al., 2022). This comparison enhances the interpretability and credibility of the selected biomarkers within the broader context of existing biological research.

## 6 Discussion

While the application of machine learning for biomarker discovery offers the potential of significant improvements over traditional statistical methods, there are still various challenges that need to be addressed before its widespread adoption. One of the major limitations that currently hampers the application of machine learning for scRNA-seq biomarker discovery is comparability. As machine learning for biomarker discovery is still in its development stage, a wide range of different strategies and algorithm metrics are currently in use across different studies. The variability of different machine learning algorithm, feature selection methods and “level” of analysis makes it difficult to directly compare findings, reproduce results, and establish a consensus benchmark. As a result, even promising biomarkers identified in one study may not be easily validated in another, limiting the reliability and widespread appeal of machine learning as a biomarker discovery method.

Additionally, the application of machine learning for scRNA-seq biomarker discovery is currently still hampered by issues of accessibility. To fully leverage machine learning for biomarker discovery, a foundational understanding of computer science and programming is required, which can be a barrier for many researchers. While this challenge is technically also present in the use of traditional statistical methods, solutions like DESeq2 and edgeR offer user-friendly packages that provide accessible tools for researchers without extensive programming expertise. This contrasts with current machine learning techniques for scRNA-seq biomarker discovery, which often is limited to paper-specific pseudocode or GitHub repositories which requires specialized knowledge to implement, thereby limiting their widespread application for biomarker discovery.

To enable broader adoption of machine learning in scRNA-seq biomarker discovery, future research should focus on establishing a consensus standard of methods through benchmarking and robust statistical evaluation to improve comparability and reproducibility. At the same time, efforts should also be made to enhance accessibility by developing standardized, user-friendly tools that lower the barrier of use for researchers without extensive computational expertise.

Details of all funding sources should be provided, including grant numbers if applicable. Please ensure to add all necessary funding information, as after publication this is no longer possible.

## References

[B1] Ashburner M. Ball C. A. Blake J. A. Botstein D. Butler H. Cherry J. M. (2000). Gene ontology: tool for the unification of biology. Nat. Genet. 25 (1), 25–29. 10.1038/75556 10802651 PMC3037419

[B2] Battiti R. (1994). Using mutual information for selecting features in supervised neural net learning. IEEE Trans. Neural Networks 5 (4), 537–550. 10.1109/72.298224 18267827

[B3] Breiman L. (2001). Random forests. Mach. Learn. 45 (1), 5–32. 10.1023/a:1010933404324

[B4] Brendel M. Su C. Bai Z. Zhang H. Elemento O. Wang F. (2022). Application of deep learning on single-cell RNA sequencing data analysis: a review. Genomics, Proteomics & Bioinforma. 20 (5), 814–835. 10.1016/j.gpb.2022.11.011 36528240 PMC10025684

[B5] Cao Y. Ghazanfar S. Yang P. Yang J. (2023). Benchmarking of analytical combinations for COVID-19 outcome prediction using single-cell RNA sequencing data. Briefings Bioinforma. 24 (3), bbad159. 10.1093/bib/bbad159 37096588 PMC10199760

[B6] Chandrashekar G. Sahin F. (2014). A survey on feature selection methods. Comput. & Electrical Engineering 40 (1), 16–28. 10.1016/j.compeleceng.2013.11.024

[B7] Chen T. Guestrin C. (2016). “Xgboost: a scalable tree boosting system,” in Proceedings of the 22nd acm sigkdd international conference on knowledge discovery and data mining.

[B8] Dong Y. Chen Z. Yang F. Wei J. Huang J. Long X. (2024). Prediction of immunotherapy responsiveness in melanoma through single-cell sequencing-based characterization of the tumor immune microenvironment. Transl. Oncol. 43, 101910. 10.1016/j.tranon.2024.101910 38417293 PMC10907870

[B9] El Naqa I. Murphy M. J. (2015). What is machine learning? Springer.

[B12] FDA-NIH Biomarker Working Group (2016). BEST (Biomarkers, EndpointS, and other Tools) Resource. Silver Spring, MD: Food and Drug Administration (United States). Available online at: http://www.ncbi.nlm.nih.gov/books/NBK326791/ (Accessed August 10, 2025). 27010052

[B10] Ferreira A. J. Figueiredo M. A. T. (2012). “Boosting algorithms: a review of methods, theory, and applications,” in Ensemble machine learning: methods and applications. Editors Zhang C. Ma Y. (New York, NY: Springer New York), 35–85.

[B11] Goel A. Mudge Z. Bi S. Brenner C. Huffman N. Giuste F. (2022). “Identification of covid-19 severity and associated genetic biomarkers based on scrna-SEQ data,” in Proceedings of the 13th ACM international conference on bioinformatics, computational biology and health informatics.

[B13] Hayakawa Y. Ozaki H. (2025). A practical guide for single-cell transcriptome data analysis in neuroscience. Neurosci. Res. 214, 9–15. 10.1016/j.neures.2025.03.006 40164433

[B14] He B. Thomson M. Subramaniam M. Perez R. Ye C. J. Zou J. (2022). CloudPred: predicting patient phenotypes from single-cell RNA-seq. Pac Symp. Biocomput. 27, 337–348. 10.1142/9789811250477_0031 34890161

[B15] Huang G.-H. Zhang Y.-H. Chen L. Li Y. Huang T. Cai Y.-D. (2021). Identifying lung cancer cell markers with machine learning methods and single-cell rna-seq data. Life 11 (9), 940. 10.3390/life11090940 34575089 PMC8467493

[B16] Kanehisa M. Goto S. (2000). KEGG: kyoto encyclopedia of genes and genomes. Nucleic Acids Res. 28 (1), 27–30. 10.1093/nar/28.1.27 10592173 PMC102409

[B17] Kang Y. Vijay S. Gujral T. S. (2022). Deep neural network modeling identifies biomarkers of response to immune-checkpoint therapy. Iscience 25 (5), 104228. 10.1016/j.isci.2022.104228 35494249 PMC9044175

[B18] Kim J. Xu Z. Marignani P. A. (2021). Single-cell RNA sequencing for the identification of early-stage lung cancer biomarkers from circulating blood. Npj Genomic Med. 6 (1), 87. 10.1038/s41525-021-00248-y 34654834 PMC8519939

[B19] Kraus V. B. (2018). Biomarkers as drug development tools: discovery, validation, qualification and use. Nat. Rev. Rheumatol. 14 (6), 354–362. 10.1038/s41584-018-0005-9 29760435

[B20] Krokidis M. G. Vrahatis A. G. Lazaros K. Skolariki K. Exarchos T. P. Vlamos P. (2023a). Machine learning analysis of alzheimer’s disease single-cell RNA-sequencing data across cortex and hippocampus regions. Curr. Issues Mol. Biol. 45 (11), 8652–8669. 10.3390/cimb45110544 37998721 PMC10670182

[B21] Krokidis M. G. Vrahatis A. G. Lazaros K. Vlamos P. (2023b). Exploring promising biomarkers for alzheimer’s disease through the computational analysis of peripheral blood single-cell RNA sequencing data. Appl. Sci. 13 (9), 5553. 10.3390/app13095553

[B22] Kursa M. B. Jankowski A. Rudnicki W. R. (2010). Boruta–a system for feature selection. Fundam. Inf. 101 (4), 271–285. 10.3233/fi-2010-288

[B23] Li W. Liu J. Zhu W. Jin X. Yang Z. Gao W. (2022). Identification of biomarkers for hepatocellular carcinoma based on single cell sequencing and machine learning algorithms. Front. Genet. 13, 873218. 10.3389/fgene.2022.873218 36353113 PMC9638064

[B24] Li H. Huang F. Liao H. Li Z. Feng K. Huang T. (2022). Identification of COVID-19-specific immune markers using a machine learning method. Front. Mol. Biosci. 9, 952626. 10.3389/fmolb.2022.952626 35928229 PMC9344575

[B25] Liberzon A. Subramanian A. Pinchback R. Thorvaldsdóttir H. Tamayo P. Mesirov J. P. (2011). Molecular signatures database (MSigDB) 3.0. Bioinformatics 27 (12), 1739–1740. 10.1093/bioinformatics/btr260 21546393 PMC3106198

[B26] Liu H. Setiono R. (1998). Incremental feature selection. Appl. Intell. 9, 217–230. 10.1023/a:1008363719778

[B27] Liu R. Dollinger E. Nie Q. (2022). Machine learning of single cell transcriptomic data from anti-PD-1 responders and non-responders reveals distinct resistance mechanisms in skin cancers and PDAC. Front. Genet. 12, 806457. 10.3389/fgene.2021.806457 35178072 PMC8844526

[B28] Lundberg S. M. Lee S.-I. (2017). A unified approach to interpreting model predictions. Adv. Neural Information Processing Systems 30. 10.48550/arXiv.1705.07874

[B29] Ma Y. Chen J. Wang T. Zhang L. Xu X. Qiu Y. (2022). Accurate machine learning model to diagnose chronic autoimmune diseases utilizing information from B cells and monocytes. Front. Immunol. 13, 870531. 10.3389/fimmu.2022.870531 35515003 PMC9065417

[B30] Mao Y. Lin Y.-Y. Wong N. K. Y. Volik S. Sar F. Collins C. (2024). Phenotype prediction from single-cell RNA-seq data using attention-based neural networks. Bioinformatics 40 (2), btae067. 10.1093/bioinformatics/btae067 38390963 PMC10902676

[B31] Miller C. Portlock T. Nyaga D. M. O’Sullivan J. M. (2024). A review of model evaluation metrics for machine learning in genetics and genomics. Front. Bioinforma. 4, 1457619. 10.3389/fbinf.2024.1457619 39318760 PMC11420621

[B32] Ng S. Masarone S. Watson D. Barnes M. R. (2023). The benefits and pitfalls of machine learning for biomarker discovery. Cell Tissue Res. 394 (1), 17–31. 10.1007/s00441-023-03816-z 37498390 PMC10558383

[B33] Nick T. G. Campbell K. M. (2007). “Logistic regression,” in Topics in biostatistics. Editor Ambrosius W. T. (Totowa, NJ: Humana Press), 273–301.

[B34] Patil A. R. Schug J. Liu C. Lahori D. Descamps H. C. Naji A. (2024). Modeling type 1 diabetes progression using machine learning and single-cell transcriptomic measurements in human islets. Cell Rep. Med. 5 (5), 101535. 10.1016/j.xcrm.2024.101535 38677282 PMC11148720

[B35] Peng H. Long F. Ding C. (2005). Feature selection based on mutual information criteria of max-dependency, max-relevance, and min-redundancy. IEEE Trans. Pattern Analysis Machine Intelligence 27 (8), 1226–1238. 10.1109/TPAMI.2005.159 16119262

[B36] Phipson B. Sim C. B. Porrello E. R. Hewitt A. W. Powell J. Oshlack A. (2022). Propeller: testing for differences in cell type proportions in single cell data. Bioinformatics 38 (20), 4720–4726. 10.1093/bioinformatics/btac582 36005887 PMC9563678

[B37] Pudjihartono N. Fadason T. Kempa-Liehr A. W. O'Sullivan J. M. (2022). A review of feature selection methods for machine learning-based disease risk prediction. Front. Bioinform 2, 927312. 10.3389/fbinf.2022.927312 36304293 PMC9580915

[B38] Qi Y. (2012). “Random forest for bioinformatics,” in Ensemble machine learning: methods and applications. Editors Zhang C. Ma Y. (New York, NY: Springer New York), 307–323.

[B39] Rainio O. Teuho J. Klén R. (2024). Evaluation metrics and statistical tests for machine learning. Sci. Rep. 14 (1), 6086. 10.1038/s41598-024-56706-x 38480847 PMC10937649

[B40] Rajula H. S. R. Verlato G. Manchia M. Antonucci N. Fanos V. (2020). Comparison of conventional statistical methods with machine learning in medicine: diagnosis, drug development, and treatment. Med. Kaunas. 56 (9). 10.3390/medicina56090455 32911665 PMC7560135

[B41] Rokach L. Maimon O. (2005). “Decision trees,” in Data mining and knowledge discovery handbook. Editors Maimon O. Rokach L. (Boston, MA: Springer US), 165–192.

[B42] Ryu Y. Han G. H. Jung E. Hwang D. (2023). Integration of single-cell RNA-seq datasets: a review of computational methods. Mol. Cells 46 (2), 106–119. 10.14348/molcells.2023.0009 36859475 PMC9982060

[B43] Saarela M. Jauhiainen S. (2021). Comparison of feature importance measures as explanations for classification models. SN Appl. Sci. 3 (2), 272. 10.1007/s42452-021-04148-9

[B44] Sabbatini G. Manganaro L. (2022). On potential limitations of differential expression analysis with non-linear machine learning models. EMBnet Journal. 28, 1035. 10.14806/ej.28.0.1035

[B45] Sarker I. H. (2021a). Machine learning: Algorithms, real-world applications and research directions. SN Comput. Sci. 2 (3), 160. 10.1007/s42979-021-00592-x 33778771 PMC7983091

[B46] Sarker I. H. (2021b). Deep learning: a comprehensive overview on techniques, taxonomy, applications and research directions. SN Comput. Sci. 2 (6), 420. 10.1007/s42979-021-00815-1 34426802 PMC8372231

[B47] Schapire R. E. (2013). “Explaining adaboost,” in Empirical inference: festschrift in honor of Vladimir N vapnik. Springer, 37–52.

[B48] St L. Wold S. (1989). Analysis of variance (ANOVA). Chemom. Intelligent Laboratory Systems 6 (4), 259–272. 10.1016/0169-7439(89)80095-4

[B49] Subramanian A. Tamayo P. Mootha V. K. Mukherjee S. Ebert B. L. Gillette M. A. (2005). Gene set enrichment analysis: a knowledge-based approach for interpreting genome-wide expression profiles. Proc. Natl. Acad. Sci. 102 (43), 15545–15550. 10.1073/pnas.0506580102 16199517 PMC1239896

[B50] Sultana A. Alam M. S. Liu X. Sharma R. Singla R. K. Gundamaraju R. (2023). Single-cell RNA-seq analysis to identify potential biomarkers for diagnosis, and prognosis of non-small cell lung cancer by using comprehensive bioinformatics approaches. Transl. Oncol. 27, 101571. 10.1016/j.tranon.2022.101571 36401966 PMC9676382

[B51] Sun X. Lin X. Li Z. Wu H. (2022). A comprehensive comparison of supervised and unsupervised methods for cell type identification in single-cell RNA-seq. Briefings Bioinforma. 23 (2), bbab567. 10.1093/bib/bbab567 35021202 PMC8921620

[B52] Suthaharan S. Suthaharan S. (2016). Support vector machine. Machine learning models and algorithms for big data classification: thinking with examples for effective learning, 207–235.

[B53] Tibshirani R. (1996). Regression shrinkage and selection via the lasso. J. R. Stat. Soc. Ser. B Stat. Methodol. 58 (1), 267–288. 10.1111/j.2517-6161.1996.tb02080.x

[B54] Vargas A. J. Harris C. C. (2016). Biomarker development in the precision medicine era: lung cancer as a case study. Nat. Rev. Cancer 16 (8), 525–537. 10.1038/nrc.2016.56 27388699 PMC6662593

[B55] Wang H. Zhang Z. Li H. Li J. Li H. Liu M. (2023). A cost-effective machine learning-based method for preeclampsia risk assessment and driver genes discovery. Cell & Bioscience 13 (1), 41. 10.1186/s13578-023-00991-y 36849879 PMC9972636

[B56] Webb G. I. Keogh E. Miikkulainen R. (2010). Naïve bayes. Encycl. Machine Learning 15 (1), 713–714.

[B57] Wenric S. Shemirani R. (2018). Using supervised learning methods for gene selection in RNA-seq case-control studies. Front. Genet. 9, 297. 10.3389/fgene.2018.00297 30123241 PMC6085558

[B58] Xie Y. Yang J. Ouyang J. F. Petretto E. (2024). scPanel: a tool for automatic identification of sparse gene panels for generalizable patient classification using scRNA-seq datasets. Briefings Bioinforma. 25 (6), bbae482. 10.1093/bib/bbae482 39350339 PMC11442147

[B59] Xiong G. Bekiranov S. Zhang A. (2023). ProtoCell4P: an explainable prototype-based neural network for patient classification using single-cell RNA-seq. Bioinformatics 39 (8), btad493. 10.1093/bioinformatics/btad493 37540223 PMC10444962

[B60] Xu Y. Goodacre R. (2018). On splitting training and validation set: a comparative study of cross-validation, bootstrap and systematic sampling for estimating the generalization performance of supervised learning. J. Analysis Test. 2 (3), 249–262. 10.1007/s41664-018-0068-2 30842888 PMC6373628

[B61] Yuan H. Zhang P. Xin Y. Liu Z. Gao B. (2024). Single cell RNA-seq identifies a FOS/JUN-related monocyte signature associated with clinical response of heart failure patients with mesenchymal stem cell therapy. Aging (Albany NY) 16 (6), 5651–5675. 10.18632/aging.205670 38517374 PMC11006470

[B62] Zhang X. Jonassen I. Goksøyr A. (2021). “Machine learning approaches for biomarker discovery using gene expression data,” in Bioinformatics. Editor Helder I. N. (Brisbane (AU): Exon Publications Copyright: The Authors). 33877765

[B63] Zhang Z. Sun C. Liu Z.-P. (2022). Discovering biomarkers of hepatocellular carcinoma from single-cell RNA sequencing data by cooperative games on gene regulatory network. J. Comput. Sci. 65, 101881. 10.1016/j.jocs.2022.101881

[B64] Zhang T. Zhao F. Lin Y. Liu M. Zhou H. Cui F. (2024). Integrated analysis of single-cell and bulk transcriptomics develops a robust neuroendocrine cell-intrinsic signature to predict prostate cancer progression. Theranostics 14 (3), 1065–1080. 10.7150/thno.92336 38250042 PMC10797290

[B65] Zhou W. Chen Y. Zheng Y. Bai Y. Yin J. Wu X.-X. (2024). Characterizing immune variation and diagnostic indicators of preeclampsia by single-cell RNA sequencing and machine learning. Commun. Biol. 7 (1), 32. 10.1038/s42003-023-05669-2 38182876 PMC10770323

